# Comparative Analysis of Vascular Structures in OLIF51 and the Lateral Corridor Approach under Supine MRI and Intraoperative Enhanced CT in the Lateral Decubitus Position

**DOI:** 10.3390/medicina60020326

**Published:** 2024-02-14

**Authors:** Yoshihisa Kotani, Hiroyuki Tachi, Atsushi Ikeura, Takahiro Tanaka, Takanori Saito

**Affiliations:** 1 Spine and Nerve Center, Department of Orthopaedic Surgery, Kansai Medical University Medical Center, Moriguchi 570-8507, Osaka, Japan; aikeike55@mac.com (A.I.); takahiro.1124.t.t@gmail.com (T.T.); 2 Department of Orthopaedic Surgery, Hokkaido University Graduate School of Medicine, Sapporo 060-0815, Hokkaido, Japan; hitachi198885@gmail.com; 3 Department of Orthopaedic Surgery, Kansai Medical University, Hirakata 573-1191, Osaka, Japan; saito@hirakata.kmu.ac.jp

**Keywords:** vascular anatomy, anterior lumbar fusion, OLIF51, lateral corridor approach

## Abstract

*Background and Objectives*: As the oblique lateral interbody fusion at L5/S1 (OLIF51) and the lateral corridor approach (LCA) have gained popularity, an understanding of the precise vascular structure at the L5/S1 level is indispensable. The objectives of this study were to investigate the vascular anatomy at the L5/S1 level, and to compare the movement of vascular tissue between the supine and lateral decubitus positions using intraoperative enhanced CT and MRI. *Materials and Methods*: A total of 43 patients who underwent either OLIF51 or LCA were investigated with an average age at surgery of 60.4 (37–80) years old. The preoperative MRI was taken to observe the axial and sagittal anatomy of the vascular position under the supine position. The intraoperative vein-enhanced CT was taken just before incision in the right decubitus position, and compared to supine MRI anatomy. Iliolumbar vein appearance and its types were also classified. *Results*: The average vascular window allowed for OLIF51 was 22.8 mm and 34.1 mm at either the L5 caudal endplate level or the S1 cephalad endplate level, respectively. The LCA was 14.2 mm and 12.6 mm at either level, respectively. The left common iliac vein moved 3.8 mm and 6.9 mm to the right direction at either level from supine to the right decubitus position, respectively. The bifurcation moved 6.3 mm to the caudal direction from supine to right decubitus. The iliolumbar vein was located at 31 mm laterally from the midline, and the MRI detection rate was 52%. *Conclusions*: The precise measurement of vascular anatomy indicated that the OLIF51 approach was the standard minimally invasive anterior approach for the L5/S1 disc level compared to LCA; however, there were many variations in quantitative anatomy as well as significant vascular movements between the supine and right decubitus positions. In the clinical setting of OLIF51 and LCA surgeries, careful preoperative evaluation and intraoperative 3D imaging are recommended for safe and accurate surgery.

## 1. Introduction

Due to the increasing aging population, minimally invasive spine surgery has gained greater attention. Classical anterior lumbosacral fusion surgery has been performed by relatively large incision with a wide surgical view due to the necessity of manipulating vascular and visceral structures [1,2]. However, recent minimally invasive technology has allowed tubular surgery with a smaller incision through either a prepsoas or a transpsoas approach [3,4]. Woods and Hynes et al. successfully minimized the conventional ALIF using a mini-retroperitoneal approach under the lateral decubitus position, which was named oblique lateral interbody fusion (OLIF) (Medtronics, Memphis, TN, USA) [3]. The advantage of anterior or anterolateral fusion includes superior anterior release, a large bone graft area, better stability and resistance to osteoporosis, and high fusion capacity [3,4,5]. However, the application of this approach for the L5/S1 level has been challenging due to the variation in major vascular tissues [4,5]. Woods and Hynes et al. successfully minimized this with standardized incision, approach protocols and the specially designed triple-arm retractor (OLIF51; Medtronics, Memphis, TN, USA), in which the vascular tissues were safely retracted and secured [3].

In turn, the use of the OLIF51 approach is sometimes disturbed due to the small vascular window. In those cases, the lateral corridor approach (LCA) can be performed, in which the approaching corridor is located between psoas muscle and ipsilateral common iliac vessels. The iliolumbar vein sometimes becomes the obstacle, and needs to be ligated.

In both the OLIF51 and LCA approaches, an understanding of the precise vascular structure at the L5/S1 level is indispensable. Specifically, the movement of vascular tissue surrounding the L5/S1 disc between the supine and lateral decubitus positions has to be taken into consideration; however, it has not been precisely reported to date. The objectives of this study were twofold. First, the precise vascular anatomy at the L5/S1 level was studied when considering both the OLIF51 and LCA surgical approaches; secondly, the movement of vascular tissue between the supine and lateral decubitus positions was quantitatively measured using intraoperative enhanced CT and MRI.

## 2. Materials and Methods

**Figure 1:**
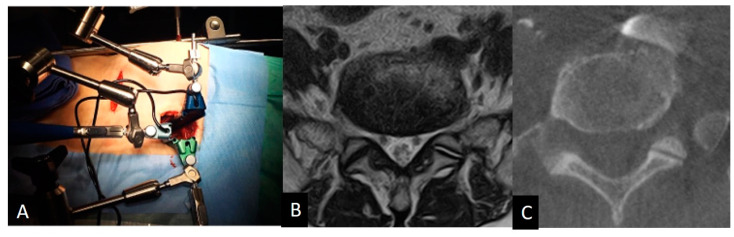
(**A**): OLIF51 surgery setup. A specially designed triple-arm retractor was used for safe retraction of vascular tissue, exposing the L5/S1 disc. (**B**) Preoperative MRI axial scan in the supine position at the L5 caudal endplate level. (**C**) Intraoperative enhanced CT scan in the right decubitus position at the L5 caudal endplate level.

Over 220 patients underwent OLIF51 or LCA surgery from 2016 to 2023, and 43 patients were included in this study. There were 16 males and 27 females with an average age at surgery of 60.4 (37–80) years old. The average body height, weight, and body mass index were 160 cm (146–187), 60.4 kg (41–86.9), and 23.5 (16.7–33.3). The disorders of patients included L5 isthmic spondylolisthesis, foraminal stenosis at L5/S1, degenerative disc disease, and pseudarthrosis of transforaminal interbody fusion at L5/S1. The infectious disease or any pathologies receiving previous retroperitoneal surgeries around lumbosacral area were excluded. All cases received preoperative lumbosacral MRI scans (Phillips Ingenia 3.0T, Canon Excelart Tiatan 1.5T) in a supine position. Before starting the OLIF51 surgery, intraoperative vein-enhanced CT (O-arm O2, Medtronics, MN, USA) was taken in the right decubitus position (Figure 1). All surgeries were performed by a single senior surgeon at a single institution.

### 2.1. Description of the OLIF51 and LCA Surgical Procedures

In the OLIF51 surgery, a 35 mm oblique incision was made two-finger medial from the anterior superior iliac spine. Following the dissection of abdominal muscles, the retroperitoneal space was enlarged and bilateral common iliac vessels were retracted. The placement of the triple-arm retractor safely secured the anterior aspect of the L5/S1 disc followed by discectomy and cartilaginous endplate removal. A 10-degree lordotic PEEK cage (MectaLIF, Medacta international, San Pietro, Switzerland) or a 12- or 18-degree lordotic PEEK cage (Sovereign, Medtronics, Memphis, TN, USA) with demineralized bone matrix (DBM) soaked with aspirated bone marrow from iliac crest was placed in the disc space. The Sovereign cage allowed the use of integrated screws for enhancing the fixation. In the posterior part of surgery, the long cortical bone trajectory screws were inserted percutaneously in the lateral decubitus position for short-segment fusion. In LCA surgery, a 35 mm oblique incision was made one-finger medial from the anterior superior iliac spine. Following the dissection of abdominal muscles, the retroperitoneal space was enlarged and the interspace between psoas muscle and common iliac vessels was approached to expose the L5/S1 disc. When the iliolumbar vein was detected, it was carefully ligated or retracted. The self-retained retractor was useful to secure the surgical view and disc preparation. After the annulotomy of the L5/S1 disc, complete nucleotomy and cartilaginous endplate removal were carried out. The 10-degree lordotic cage was placed with DBM soaked with aspirated bone marrow from iliac crest. In both approaches, a meticulous layer muscular suture was carried out to prevent abdominal wall herniation. All surgeries were conducted with the use of surgical navigation and O-arm (Stealthstation 7, Medtronics, Memphis, TN, USA).

### 2.2. Evaluation of Vascular Anatomy in the Supine Position on MRI

**Figure 2:**
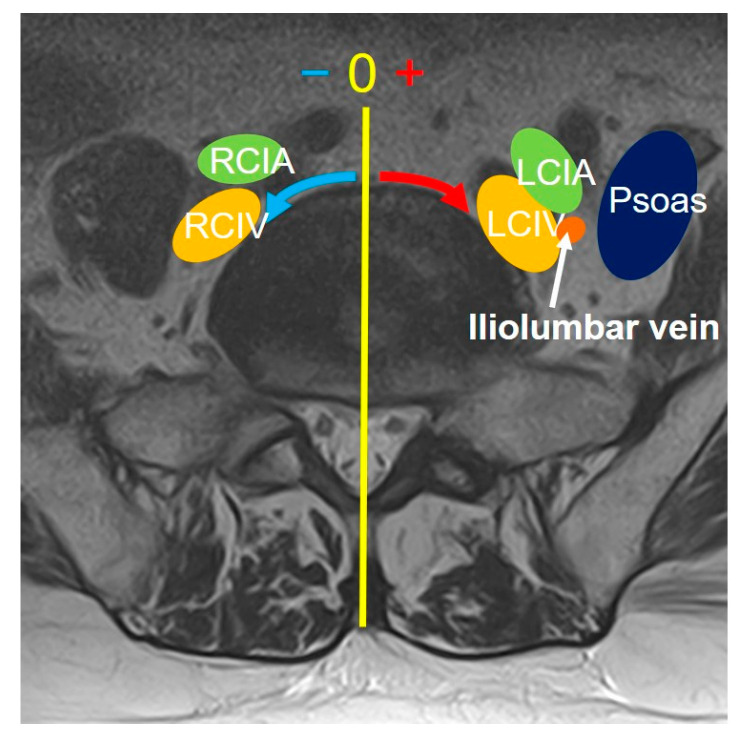
Measurement of the vascular position at the L5/S1 disc level. The distance from the midline of the disc to each vascular tissue was measured either counterclockwise (minus) or clockwise (plus). RCIA: right common iliac artery; RCIV: right common iliac vein; LCIA: left common iliac artery; LCIV: left common iliac vein; Psoas: iliopsoas muscle.

**Figure 3:**
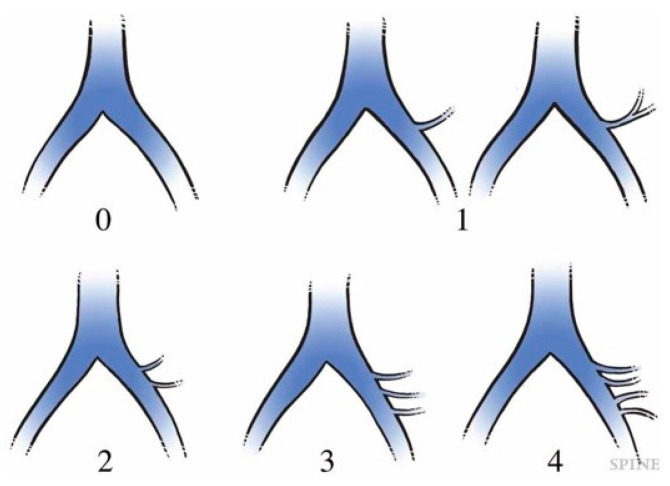
Classification of the iliolumbar vein according to Nalbandian et al. [6].

**Figure 4:**
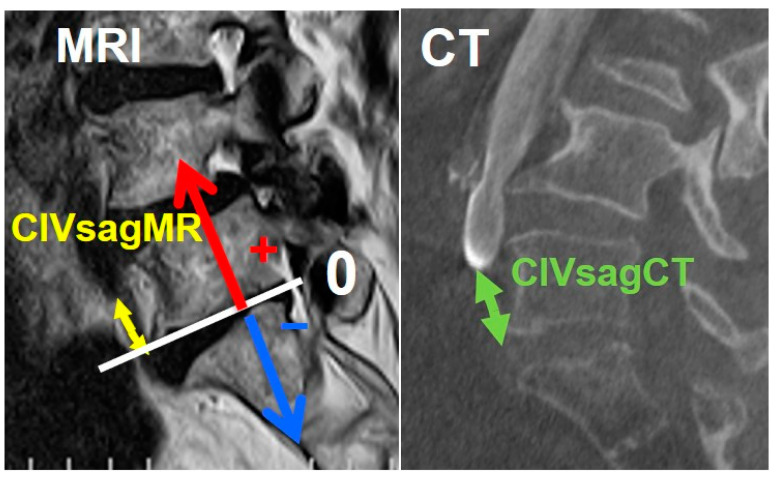
Measurement of common iliac bifurcation either on MRI or CT. The position was measured from the L5 caudal endplate to the bifurcation and expressed as plus (cephalad) or minus (caudal). CIVsagMR: common iliac vein in sagittal MRI; CIVsagCT: common iliac vein in sagittal CT.

On MRI axial scan at either the caudal L5 endplate level or the cephalad S1 endplate level, the distance from the sagittal mid-vertebral line to border of the right and left common iliac vessels was measured as (L5-LCIV, L5-RCIV, L5-LCIA, L5-RCIA, S1-LCIV, S1-RCIV, S1-LCIA, S1-RCIA) (Figure 2). The vascular window was measured between right and left iliac vessels, expressed as the central corridor window (CCW). The lateral corridor window was expressed as the distance between the lateral border of the left common iliac vein and medial border of psoas muscle (LCW) (Figure 2). Iliolumbar vein anatomy was also evaluated in terms of the visualization rate (%), the axial position from the sagittal mid-vertebral line (L5-ILV, S1-ILV). The type of iliolumbar vein was classified according to Nalbandian et al. [6] (Figure 3). Type 0 refers to no ILV, type 1 is the single variant. Multiple ILVs are categorized into types 2, 3, and 4 according to the number of veins found. The vertical position of common iliac vein bifurcation was measured from the lower endplate of L5 (Figure 4). For precise measurements, all imaging data were transferred to the three-dimensional image analysis system (Synapse Vincent, FujiFilm, Co., Tokyo, Japan), and the measurements were performed on its workstation.

### 2.3. Intraoperative Evaluation of Vein Anatomy in the Right Decubitus Position on Enhanced CT

The intraoperative vein anatomy in the right decubitus position was evaluated with vein-enhanced intraoperative CT (O-armO2, Medtronics) (Figure 1C). Prior to the incision made for OLIF51 surgery, the contrast medium was injected from the peripheral vein of left foot, and a O-arm 3D scan was subsequently taken. The axial and sagittal positions of the vein structure surrounding the L5/S1 disc were measured according to the same parameters as those in the supine position on MRI (Figure 2). The vein position on intraoperative CT was subsequently compared to that in the supine position on MRI, and the movement of the vein structure was quantitatively analyzed (Figure 2 and Figure 4).

This study was conducted based on the acceptance of the university ethics committee, and patient data and radiographs without personal information in this report are shown with the consent of the patients.

## 3. Results

### 3.1. Evaluation of Vascular Anatomy in the Supine Position

**Figure 5:**
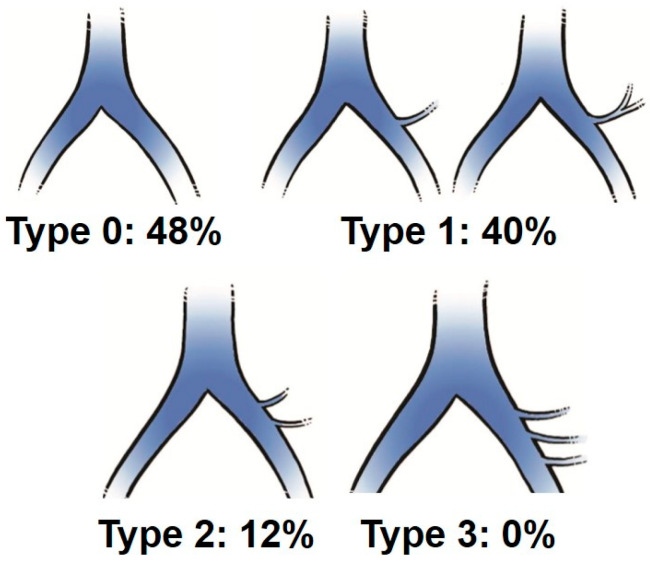
Results of the iliolumbar vein observation on MRI in the present study.

The average positions of right and left CIV were −22.2 mm (SD:4.1 mm) and 8.2 mm (4.0) at the L5 lower endplate level, and −27.9 mm (2.9) and 16 mm (4.4) at the S1 upper endplate level, respectively (Table 1). The average positions of right and left CIA were −17.9 mm (4.5) and 17.8 mm (5.7) at the L5 lower endplate level, and −21.3 mm (4.0) and 25.1 mm (5.3) at the S1 upper endplate level, respectively. The average CCWs were 22.8 mm (7.3) at the L5 lower endplate level, and 34.1 mm (7.3) at the S1 upper endplate level. The average bifurcation position from the L5 lower endplate was 25.3 mm (7.0 mm SD). The average LCWs were 14.2 mm (4.8) at the L5 lower endplate level, and 12.6 mm (3.6) at the S1 upper endplate level. The visualization rate (%) of the left iliolumbar vein (ILV) on preoperative MRI was 52% and the type was categorized into type 0 (48%), type 1 (40%), type 2 (12%), and type 3 (0%) (Figure 5). The average position of the iliolumbar vein was 31.1 mm (5.7) at the L5 lower endplate level, and 30.9 mm (8.1) at the S1 upper endplate level (Table 1).

### 3.2. Intraoperative Evaluation of Vein Anatomy in the Right Decubitus Position

From the measurement of vein position on intraoperative enhanced CT, axial and sagittal movement of the vascular structure were summarized in Table 2. From the position change in supine to right decubitus, right and left CIV moved to 0.8 mm and 3.8 mm to the right at the L5 lower endplate level, respectively (Table 2). At the S1 upper endplate level, left CIV moved 6.9 mm to the right, and right CIV moved 0.3 mm to the left. Overall, left CIV moved to the right much more than right CIV. The CCW at the L5 lower endplate level became 3.6 mm smaller than that in the supine position. The CCW at the S1 upper endplate level became 2.5 mm smaller than that in the supine position. The vein bifurcation level moved 6.3 mm caudally when compared to the supine position.

**Figure 6:**
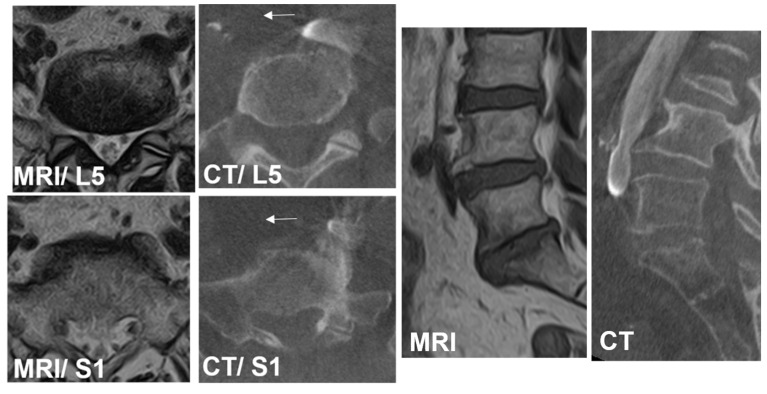
A 75-year-old woman with L4 degenerative spondylolisthesis and L5/S1 degenerative disc disease received L4-S1 OLIF followed by lateral position percutaneous screwing and fixation. The comparison between preoperative supine MRI images and intraoperative vein-enhanced CT demonstrated left CIV movement of 6.7 mm to the right in the right decubitus position at the L5 lower endplate level, and 3.4 mm to the right at the S1 upper endplate level (arrows). The vein bifurcation moved 8.5 mm to the caudal direction under the right decubitus position.

**Case presentation**
Case 1: A 75-year-old woman with L4 degenerative spondylolisthesis and L5/S1 degenerative disc disease received L4-S1 OLIF followed by lateral position percutaneous screwing and fixation. The comparison between preoperative supine MRI images and intraoperative vein-enhanced CT demonstrated left CIV movement of 6.7 mm to the right in the right decubitus position at the L5 lower endplate level, and 3.4 mm to the right at the S1 upper endplate level. The vein bifurcation moved 8.5 mm to the caudal direction under the right decubitus position (Figure 6).

Case 2: 76 years old, female, pseudarthrosis of L5/S1 transforaminal interbody fusion.

**Figure 7:**
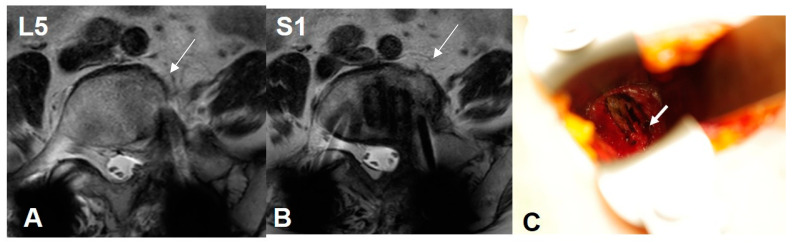
Case 2: 76 years old, female, pseudarthrosis of L5/S1 transforaminal interbody fusion. The preoperative CCW was 7.4 mm and 9.6 mm at the L5 and S1 levels, respectively. The lateral corridor approach was conducted instead of the OLIF51 approach (arrows) (**A**,**B**). The iliolumbar vein was ligated successfully (**C**).

The preoperative CCW was 7.4 mm and 9.6 mm at the L5 and S1 levels, respectively. The lateral corridor approach was conducted instead of the OLIF51 approach (Arrows). The iliolumbar vein was ligated and interbody fusion was conducted successfully (Figure 7).

## 4. Discussion

The preoperative evaluation for vascular structures is indispensable for the safe and accurate minimally invasive anterior approach to the lumbosacral spine. The supine MRI or enhanced CT scan are often used for the evaluation; however, the vascular and visceral structures move downwards under an OLIF51 or LCA approach in the lateral decubitus position. Deukmedjian et al. evaluated the movement of abdominal structures on MRI during the supine and right decubitus positions [7]. They found some movement of aorta, vena cava, and kidney in the lateral position, but the precise vascular anatomy with reference to the disc was not demonstrated at the L5/S1 level. The present study was the first to demonstrate the precise vascular anatomy in both the OLIF51 and LCA approaches as well as its movement under the change between the supine and lateral decubitus positions.

There were some studies reporting a vascular window (CCW) that allowed for the OLIF51 approach at the L5/S1 disc level [8,9] (Table 3). Choi et al. measured the CCW at the mid-disc level on MRI, which was 27 mm on average (8–46) [8]. Nagamatsu et al. utilized combined MRI and CT images and reported 29.7 mm at the L5 caudal endplate level, and 36.9 mm at the S1 cephalad endplate level [9]. The present study showed 22.9 mm and 34.1 mm at the L5 caudal and S1 cephalad endplate levels, respectively. Our results were smaller in both levels in spite of the larger body height compared to Nagamatsu’s report.

There have been three previous reports demonstrating the length from the midline to left CIV at the L5/S1 level [10,11,12] (Table 3). Liu et al. utilized CT angiography and reported a length of 15.9 mm on average [10]. Davis et al. reported a similar length of 14.75 mm in the cadaveric measurement [11]. In turn, Molinares et al. reported a smaller value of 10 mm on average measured on MRI [12]. Our data showed relatively smaller values of 8.7 mm and 16.0 mm at the L5 caudal and S1 cephalad endplate levels, respectively.

The bifurcation level of the common iliac vein was reported by Davis and Nagamatsu [9,11]. Davis et al. reported a 23.8 mm cephalad from the L5 caudal endplate level, and Nagamatsu et al. reported a 23.7 mm cephalad. Our result was 25.3 mm, which was almost equivalent to other reports. The LCW length was reported by Molinares et al. as 10.8 mm on average [12]. The present study demonstrated 14.3 mm on average, reflecting the smaller CCW and larger LCW in our series.

In the present study, we found the iliolumbar vein in 52% on preoperative MRI scan. Nalbandian et al. examined the detailed anatomy of ILV in 159 cases of anterior lumbar open surgeries, and only 1.3% were missing the LIV [6]. As the LIV systems were difficult to visualize clearly on either MRI or enhanced CT [6,13], our detection rate of 52% on MRI was relatively reasonable. In our evaluation, type 1 and type 2 were detected in 40% and 12%, respectively (Figure 5). In Nalbandian’s report, 73% showed a single branch of type 1, and 17% showed double branches of type 2 [6]. We speculate that 48% of our type 0 may have the possibility to show ILVs that we actually expose in the operation field.

Using intraoperative enhanced CT, we quantitated the movement of vascular tissues from the supine to right decubitus position when compared to preoperative supine MRI. As shown on Table 2, there have been discrepancies of CIV movement between the right and left sides. The left CIV moved more to the right side, by 3.8 mm and 6.9 mm, at the L5 caudal and S1 cephalad endplate levels, respectively. This led to a 3.6 mm and 2.5 mm decrease in the vascular window at the L5 caudal and S1 cephalad endplate levels, respectively. Choi et al. compared the vascular window between the supine and right decubitus positioned MRI, showing a 5.2 mm decrease in the window [8]. We examined the intraoperative anatomy in actual operative position under general anesthesia settings. In our OLIF51 surgery, the hip and knee were extended for more caudal incision to fit the sacral slope, so the vascular tension may increase in our series. The vein bifurcation moved caudally by 6.3 mm from the supine to the right decubitus position in our study. This was beyond our expectations and this also contributed to the narrowing of vascular window for the OLIF51 approach.

The quantitative data demonstrated in this study will be useful for minimally invasive OLIF51 and LCA. The present study showed that the average vascular window for the OLIF51 approach was 23–34 mm, and it was 12–14 mm for LCA. This meant that the average vascular anatomy at L5/S1 required the OLIF51 approach instead of LCA; however, there was wide variation in the CCW, from 0 to 63 mm, in our study. Liu et al. described that a vascular window below 10 mm was impractical for OLIF51 surgery [10]. Choi et al. detailed that in patients with a vascular window below 15 mm and an absence of perivascular fat, extreme caution should be taken when using the OLIF51 approach [8]. We recommend the OLIF51 approach for a vascular window over 15 mm; however, there are many other important factors that needed to be considered. First, OLIF51 surgery basically require the mobility of left CIV (LCIV), and adhesion between CIV and the disc is a major limiting factor. When there is much perivascular fat, good mobility is anticipated. However, when perivascular tissue is scarce, or LCIV is directly attached to the osteophyte, vascular mobility may be limited. Chung et al. classified the relationship between LCIV and the L5/S1 disc into three categories: type I: LCIV located at the lateral one-third of the disc or floating from the disc; type II: medial two-third, but LCIV surrounded by perivascular fat; type III: no perivascular fat [14]. Their data demonstrated that 49.2% was type I, 27.7% was type II and 23.1% was type III. Five out of seven vascular injury cases occurred in type III and should be avoided. According to my OLIF51 experience over 220 cases, even in type III cases, LCIV mobility was mostly obtained during surgery. For successful retraction, the step-by-step retraction of LCIV and extensive disc release should proceed before extending the window. Secondly, surgical indication should be considered carefully. Specifically, patients who received previous lateral interbody or transforaminal interbody fusion at the cephalad segment sometimes showed adhesions around the approaching plane. As is obvious in the previous malignancy surgery of the retroperitoneal area, even patients who underwent intra-abdominal surgery sometimes showed adhesions in the retroperitoneal area. Therefore, the precise examination of past history and alternative cages or approaches should always be prepared. Based on previously described factors, our present exclusion criteria for the OLIF51 approach is as follows: (1) a vascular window <15 mm; (2) a previous history of malignancy surgery around the area anterior close to the promontorium; (3) severe adhesion suspected due to previous anterior surgery, osteophyte, or anterior longitudinal ligament ossification; (4) vascular anomaly; and (5) a segment being ineffective by indirect neural decompression due to ligamentum flavum calcification.

There were several advantages and limitations in this study. Although several studies investigated the vascular anatomy of the OLIF51 or LCA approach, they utilized the cadaver or laterally positioned MRI [8,9,11]. The present study measured the vascular anatomy in the actual operative setting of OLIF51 surgery in the right decubitus position using intraoperative enhanced CT (O-arm). This minimized the error caused by the patient not being positioned in the true lateral position on MRI, or muscular tonus changing the vascular position without general anesthesia. There was a technical limitation in which the measurement of the vascular position was performed between supine MRI and enhanced CT. In a usual OLIF51 surgery setting, the author firstly takes a MRI scan for vascular and neural observation. If the vascular window is very small, we add the enhanced CT, so most cases only received MRI without additional radiation exposure. To accurately compare both modalities, we utilized the three-dimensional imaging system to set the gantry parallel to the L5 caudal or S1 cephalad endplate, and measured the vascular position precisely.

## 5. Conclusions

The precise measurement of vascular anatomy indicated that the OLIF51 approach was the standard minimally invasive anterior approach for the L5/S1 disc level compared to LCA; however, there were many variations in quantitative anatomy as well as significant vascular movements between the supine and right decubitus positions. In the clinical setting of OLIF51 and LCA surgeries, careful preoperative evaluation and intraoperative 3D imaging are recommended for safe and accurate surgery.

## Figures and Tables

**Table 1:** The vascular position and window in the supine position.

	CIV	CIA	CCW	LCW	ILV
L5 Right	−22.2 (4.1)	−17.9 (4.5)	22.8 (7.3)	14.2 (4.8)	31.1 (5.7)
L5 Left	8.2 (4.0)	17.8 (5.7)			
S1 Right	−27.9 (2.9)	−21.3 (4.0)	34.1 (7.3)	12.6 (3.6)	30.9 (8.1)
S1 Left	16.0 (4.4)	25.1 (5.3)			

The average and standard deviation are shown (mm). Negative value mean the distance to the right direction from the sagittal mid-central line of vertebral endplate in the axial scan. CIV: common iliac vein, CIA: common iliac artery, CCW: central corridor window, LCW: lateral corridor window, and ILV: Iliolumbar vein.

**Table 2:** Vascular movement and window change in the right decubitus position.

	CIV	CCW	Vein Bifurcation
L5 Right	−0.8 (2.3)	−3.6 (2.7)	6.3 (2.3)
L5 Left	−3.8 (3.1)		
S1 Right	0.3 (2.2)	−2.5 (1.6)	
S1 Left	−6.9 (3.0)		

The average and standard deviation are shown (mm). A negative value means the vascular movement of CIV to the right direction in the axial scan. The reduction in CCW is expressed as a negative value. Vein bifurcation movement to the caudal direction is expressed as a positive value. CIV: common iliac vein; CCW: central corridor window.

**Table 3:** Comparison of vascular measurement data.

	Modality	Mean (mm)	SD (mm)	Max (mm)	Min (mm)
**CCW**					
This study (L5 caudal)	MRI	22.9	7.3	63	0
This study (S1 cephalad)	MRI	34.1	7.3	59.7	6.8
Choi (Mid disc) [8]	MRI	27	9.4	46	8
Nagamatsu (L5 caudal) [9]	MRI/CT	29.7	10.7		
Nagamatsu (S1 cephalad) [9]	MRI/CT	36.9	10.3		
**Midline to Lt CIV**					
This study (L5 caudal)	MRI	8.7	5.4	21.4	0
This study (S1 cephalad)	MRI	16.0	4.4	31.3	−6.5
Liu [10]	CT angiography	15.9	9.3	30.2	0
Davis [11]	Cadaver	14.75	6.90	25.0	0
Molinares [12]	MRI	10.0	8.3	28.4	0
**IVC bifurcation from L5 caudal**					
This study	MRI	25.3	14.1	59.6	0
Davis [11]	Cadaver	23.8	13.3	58.0	10.0
Nagamatsu [9]	MRI/CT	23.7	10.9		
**LCW**					
This study (L5 caudal)	MRI	14.3	9.6	38.5	3.0
Molinares [12]	MRI	10.8	9.2	24.6	4.6

Mean, standard deviation (SD), max (Maximum), and min (minimum) values are shown. CCW: central corridor window; CIV: common iliac vein; LCW: lateral corridor window.

## Data Availability

The data presented in this study are available in this article.
